# Studying the Thermodynamic Phase Stability of Organic–Inorganic Hybrid Perovskites Using Machine Learning

**DOI:** 10.3390/molecules29132974

**Published:** 2024-06-22

**Authors:** Juan Wang, Xinzhong Wang, Shun Feng, Zongcheng Miao

**Affiliations:** 1 Xi’an Key Laboratory of Advanced Photo-Electronics Materials and Energy Conversion Device, School of Electronic Information, Xijing University, Xi’an 710123, China; wangjuan@xijing.edu.cn (J.W.); fengshun@xijing.edu.cn (S.F.); 2 School of Artificial Intelligence, Optics and Electronics (iOPEN), Northwestern Polytechnical University, Xi’an 710072, China

**Keywords:** organic–inorganic hybrid perovskites, thermodynamic phase stability, machine learning, LightGBM algorithms

## Abstract

As an important photovoltaic material, organic–inorganic hybrid perovskites have attracted much attention in the field of solar cells, but their instability is one of the main challenges limiting their commercial application. However, the search for stable perovskites among the thousands of perovskite materials still faces great challenges. In this work, the energy above the convex hull values of organic–inorganic hybrid perovskites was predicted based on four different machine learning algorithms, namely random forest regression (RFR), support vector machine regression (SVR), XGBoost regression, and LightGBM regression, to study the thermodynamic phase stability of organic–inorganic hybrid perovskites. The results show that the LightGBM algorithm has a low prediction error and can effectively capture the key features related to the thermodynamic phase stability of organic–inorganic hybrid perovskites. Meanwhile, the Shapley Additive Explanation (SHAP) method was used to analyze the prediction results based on the LightGBM algorithm. The third ionization energy of the B element is the most critical feature related to the thermodynamic phase stability, and the second key feature is the electron affinity of ions at the X site, which are significantly negatively correlated with the predicted values of energy above the convex hull (E_hull_). In the screening of organic–inorganic perovskites with high stability, the third ionization energy of the B element and the electron affinity of ions at the X site is a worthy priority. The results of this study can help us to understand the correlation between the thermodynamic phase stability of organic–inorganic hybrid perovskites and the key features, which can assist with the rapid discovery of highly stable perovskite materials.

## 1. Introduction

Organic–inorganic hybrid perovskites, comprising both organic and inorganic components, exhibit promising prospects for applications in photovoltaic power generation, luminescence, ferroelectricity, optical detection, and other fields [1,2]. Particularly in the field of solar cells, they offer advantages such as low production costs, minimal environmental impact, and high photoelectric conversion efficiency [3,4]. However, the commercialization process of perovskite solar cells has been impeded by their inherent instability [5,6]. The stability of these cells is primarily influenced by the structural stability of perovskite materials [7]. The structure of organic–inorganic hybrid perovskite materials is susceptible to damage in high-temperature or high-humidity environments, resulting in a layered perovskite structure [8]. Some perovskite materials are metastable at room temperature, and if they are exposed to moisture, heat, light, or polar solvents, the phase transition of perovskites can be accelerated, which prevents their application in solar cells [9,10]. Consequently, developing highly stable organic–inorganic perovskites holds significant potential for facilitating the commercial application of perovskite solar cells. However, identifying stable perovskite materials from the thousands of available perovskite materials presents significant challenges. Their stability is influenced by numerous complex factors, which cannot be accurately described using traditional theoretical models or empirical rules [11]. Therefore, identifying key factors that affect the stability of perovskite materials can provide crucial guidance for the research and development of highly stable perovskites.

The stability of perovskites has mainly been studied in terms of their structural stability and thermodynamic phase stability at present. The structural stability of perovskites can be researched using the tolerance factor. In [12], Bartel et al. developed an accurate, physically interpretable, and one-dimensional tolerance factor to predict the stability of perovskite oxides and halides, where the stability refers to the stability of the perovskite lattice structure. The thermodynamic phase stability is a key parameter that broadly governs whether the material is expected to be synthesizable, and whether it may degrade under certain operating conditions [13]. The energy above the convex hull (E_hull_) provides a direct measure of the thermodynamic phase stability [14]. Meanwhile, Wanjian Yin found that there is no significant quantitative relationship between the thermodynamic stability of cubic perovskite and the tolerance factor [15]. Works on the evaluation of perovskite thermodynamic stability are important for validating the results recorded when researching perovskite stability according to the tolerance factor [16]. The E_hull_ value can be calculated using density functional theory (DFT). A greater positive value of E_hull_ indicates a lower stability [17]. However, the huge computational cost limits the use of DFT in large chemical spaces [18]. Compared with DFT calculations, the utilization of machine learning in material development offers significant advantages. It can effectively reduce the expenditure on research and development, expedite the process of material development, and enhance material performance, thereby presenting a promising prospect for the advancement of novel materials [19,20]. The machine learning (ML) method relies on experimental and computational data as the basis for learning [21]. By analyzing a large amount of test data, machine learning can identify key factors affecting the stability of materials and provide a sensitivity analysis to help optimize the preparation process of materials [22]. For example, machine learning models are trained using data on the structure and performance of known materials, which allows them to learn and establish relationships between the structure and performance of materials so as to predict and screen the materials with excellent properties [23]. Machine learning can simulate and calculate the materials of different components, analyze their advantages and disadvantages, and realize the optimization of components [24]. It can also analyze the optimal preparation conditions through various aspects of the material preparation process, which can guide the acquisition of optimal materials [25,26].

Since machine learning shows great potential for applications in material development, more and more scholars have applied it in the prediction of perovskite materials. Jie Zhao et al. [27] constructed classification and regression models to predict the thermodynamic stability and energy above the convex hull. They found that the highest occupied molecular orbital energy and the elastic modulus of the B-site elements for perovskite oxides are the top two features for stability prediction. Liu et al. [28] successfully classified 37 thermodynamically stable materials and 13 metastable oxide perovskites based on the E_hull_ value. Talapatra et al. [29] focused on the detailed chemical space of mono-oxide perovskites and di-oxide perovskites and optimally explored this space to identify stable compounds that might form using a random forest generation ML model. Yunlai Zhu et al. [30] developed an optimal model for predicting the thermodynamic stability of perovskites using machine learning based on a data set of 2877 ABX_3_ and found that the extreme gradient-boosting regression technique showed the best performance in predicting the E_hull_ value, with an RMSE of 0.108 and an R^2^ of 0.93. Bhattacharya et al. [31] revealed the key structural features that govern ABO_3_-type oxide perovskites and the extent of correlation between the stability and formability of these compounds using machine learning. So, ML has applicability in the prediction of perovskite stability and has great potential to help accelerate the screening of high-stability perovskite materials.

For organic–inorganic hybrid perovskites (HOIPs), scholars have studied the band gap prediction of organic–inorganic perovskites [32], and the classification of two-dimensional and three-dimensional organic–inorganic perovskites [33] used in ML methods. Zhang et al. [19] proposed an interpretive ML strategy to speed up the discovery of potential HOIPs, which employed 102 samples as a training set. Yiqiang Zhan et al. pursued superior efficiency and thermal stability by combining machine learning with high-throughput computing to accelerate the discovery of hybrid organic–inorganic perovskites A_2_BB’X_6_ from a large number of candidates [34]. However, there are relatively few research projects on predicting the thermodynamic phase stability of organic–inorganic perovskites based on machine learning and exploring the relationship between thermodynamic phase stability and the features of elements in perovskites.

So, in this work, four methods, random forest regression (RFR), support vector machine regression (SVR), XGBoost regression, and LightGBM regression, were used to establish the prediction model of organic–inorganic hybrid perovskites to predict the energy above the convex hull (E_hull_) value. At the same time, the Shapley Additive Explanation (SHAP) model was used to analyze the prediction results to help understand the correlation between the thermodynamic phase stability prediction and key features of organic–inorganic hybrid perovskites.

## 2. Results and Discussion

### 2.1. Feature Engineering

#### 2.1.1. Feature Processing

The data set was preprocessed by the exploratory data analysis. By utilizing MinMaxScaler for feature scaling, the disparity in scales among features was mitigated, thereby promoting a more equitable distribution of weights across different features and enhancing both the performance and stability of the model [35]. The scaling of features can expedite the convergence process of the optimization algorithm and enhance the efficiency of model training. The MinMaxScaler operated based on the following principles: 

(1) Found the minimum (*min*) and maximum (*max*) for each feature in the training set; 

(2) Calculated, for each sample, the normalization result of its eigenvalue by the formula:

(1) $$X_{normalize}=(x-min)/(max-min);$$

(3) The value of each feature was mapped to the interval of [0, 1]. 

**Figure 1:**
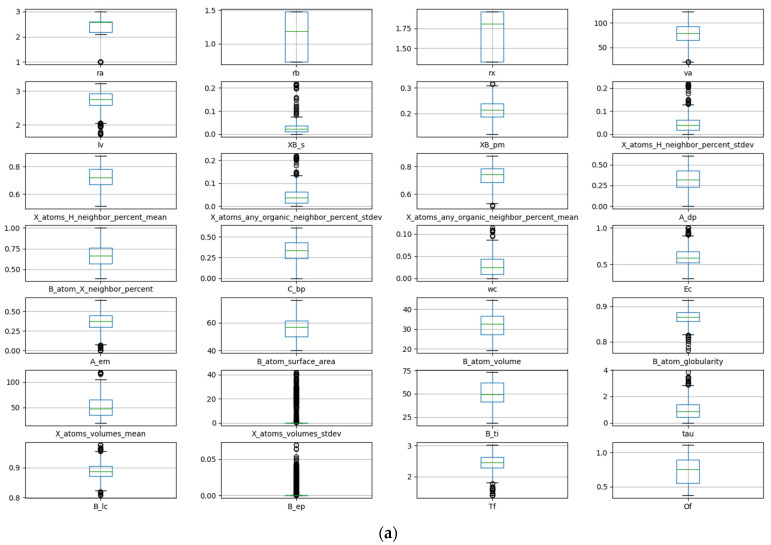

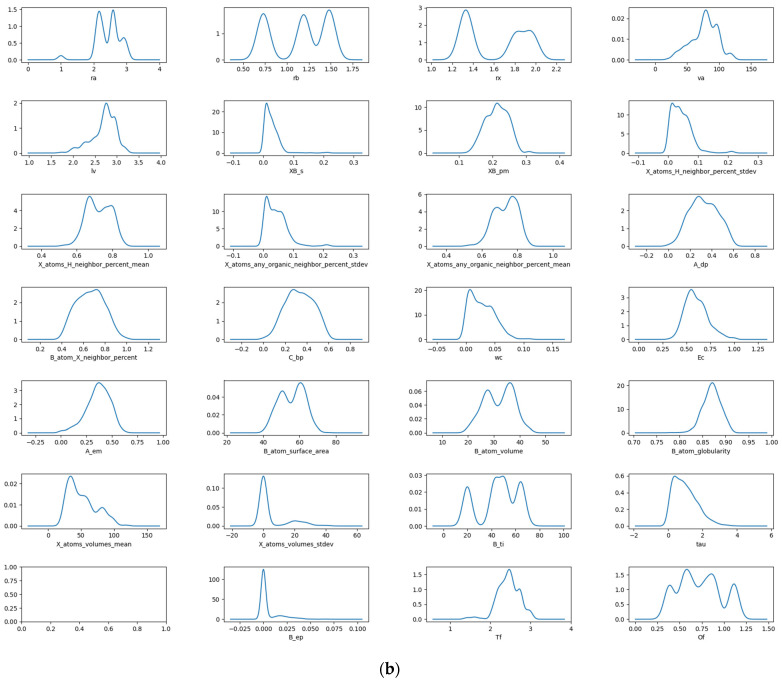
Box plot (**a**) and density map (**b**) of features about E_hull_.

The data are visualized and analyzed using a box plot and density map. The distribution and outlier of the data can be judged by observing the position and size of the box, the length of the longitudinal line, and the outlier points. Figure 1a shows the box plot of stability data. It can be observed that there are obvious outliers in the data of seven descriptors: crystal length, standard deviation of proportion for B and X atoms, percentage standard deviation of adjacent atoms in X position, sphericity of the B atom, standard deviation of the volume for atom at X position, and crystal structure at B element. The removal of these anomalous data is necessary during the process of feature manipulation. The density diagram of the data set about stability is shown in Figure 1b. The distribution and skewness of the original data in the data set can be assessed by examining the density curve. The majority of the density curves exhibit a unimodal symmetric distribution, implying a uniform data distribution. The density curves of these two descriptors, crystal structure at B element and standard deviation of the volume for an atom at X position, show left or right skew, indicating that there is skew in the data. The density curves of the radius of the B-site ion, the radius of the A-site ion, and the third ionization energy of the B element have multi-peak distributions, suggesting the presence of diverse data distributions.

#### 2.1.2. Feature Correlation Analysis

The technique of feature selection plays a pivotal role in the field of machine learning. The main purpose is to select the most relevant or representative subset of features from the raw data in order to effectively reduce the dimension of data and improve the performance of the model. It can help weed out useless or redundant features, make the model easier to understand and interpret, and improve the accuracy and generalization of the model. In feature selection, the Pearson correlation coefficient [36] is used to evaluate the correlation between each feature and the target variable, and the features that exhibit a strong correlation with the target variable are selected. The Pearson correlation coefficient is a statistic that measures the strength of the linear relationship between two variables, and it is often used to assess the correlation between two features. Its definition is shown in Formula (2).

(2) $$\rho_{X,Y}=\frac{cov(X,Y)=1/(n-1)\sum_{n}^{i=1}(X_{i}-X^{-})(Y_{i}-Y^{-})}{\sigma_{X}\sigma_{Y}}$$

**Figure 2:**
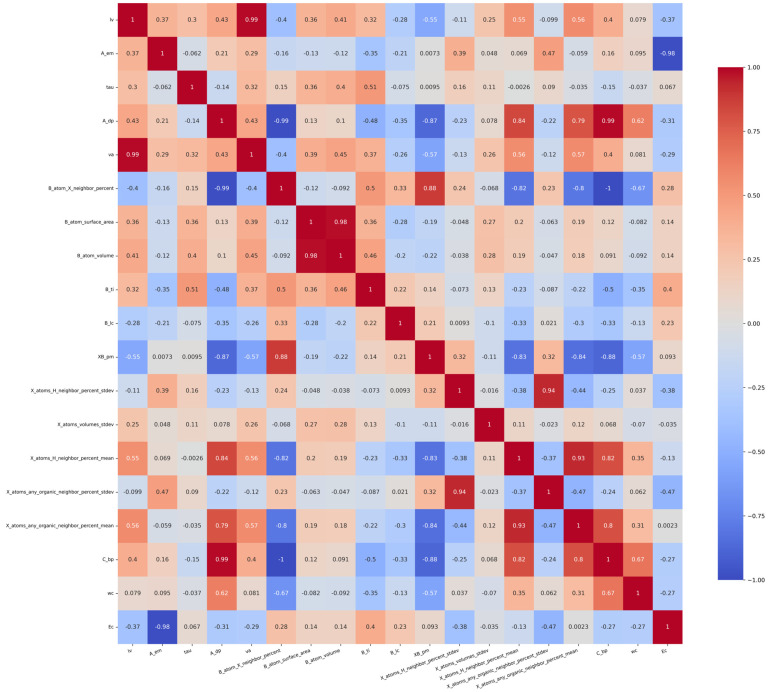
Pearson correlation coefficients of features about E_hull_.

The value of the Pearson correlation coefficient ranges from −1 to 1, where −1 means a complete negative correlation, 1 means a complete positive correlation, and 0 means no linear correlation. Firstly, unnecessary features are eliminated to reduce the dimension of the data set. In order to eliminate the influence of unnecessary features on the accuracy of the prediction model, 28 features whose correlation coefficient is close to 0 were excluded. The data set was initially screened by the derived correlation coefficient. The correlation coefficient among the ra, rb, B_ep, Tf, Of, X_atoms_volumes_mean, X_atoms_volumes_stdev, B_atom_globularity, XB_s, and rx is all in the range of 0–0.2, indicating that these features are not correlated. Therefore, the above features are not taken into consideration in predicting E_hull_. We removed irrelevant features and retained the related features of lv, A_em, tau, A_dp, va, B_atom_X_neighbor_percent, B_atom_surface_area, B_atom_volume, B_ti, B_lc, XB_pm, and X_atoms_H_neighbor_percent_stdev, X_atoms_volumes_stdev, X_atoms_H_neighbor_percent_mean, X_atoms_any_organic_neighbor_percent_stdev, X_atoms_any_organic_neighbor_percent_mean, C_bp, wc, and Ec. The Pearson correlation coefficient of these features is shown in Figure 2. The gradient color bar on the right corresponds to the magnitude of the correlation coefficient. The color red indicates a positive correlation, while blue signifies a negative correlation; furthermore, the intensity of the color corresponds to the strength of the correlation.

As shown in Figure 2, when the Pearson correlation coefficient between two features is close to 1, it means that when one feature increases, the other feature also increases in almost the same proportion, which usually causes the problem of multicollinearity. The presence of multicollinearity can have a detrimental impact on the performance of linear models that rely on estimated coefficients. In this case, the model’s stability may be compromised, leading to unreliable estimates of coefficients and diminished interpretability. For example, the correlation coefficient between va and lv is as high as 0.99, indicating that the two features have a high linear correlation. In order to reduce the dimension of data, consider removing one of these highly correlated features. The method of deleting highly correlated features is as follows: when the Pearson correlation coefficient of two features A and B is close to 1, delete any one of the features, construct the feature set separately, and perform the prediction. Compare the R^2^ values of the prediction model between the two cases of feature A deleted and feature B deleted. Select the case with a high R^2^. At the same time, MSE and MAE should also be considered. After deleting one of the highly correlated features, MSE is used to determine whether the model would be over-fitting or under-fitting. In addition, it is also necessary to consider key characteristics affecting thermodynamic stability, such as va. Although va and lv are highly correlated, the chemical lattice constant va is strongly related to the stability of the compound. So, the va feature is preserved.

**Figure 3:**
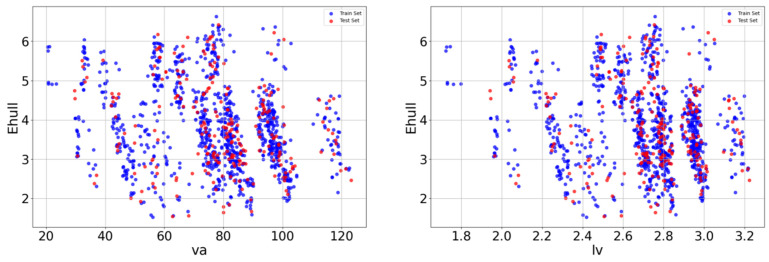
Relationship between va and lv characteristics and E_hull_.

After the Pearson correlation analysis, redundant features with particularly high linear correlations were screened out and scatter plots were drawn between crystal length, crystal volume, and target variables, as shown in Figure 3. The diagram shows the relationship between the two variables and E_hull_. In the scatter plot, each data point represents a sample, blue is the point of the training set, red is the point of the test set, and its *x*-axis and *y*-axis coordinates represent the values of the two variables, respectively. By observing the distribution and trend of data points in the scatter plot, it is found that the relationship between va and lv and E_hull_ has a nonlinear relationship.

#### 2.1.3. Feature Screening

**Figure 4:**
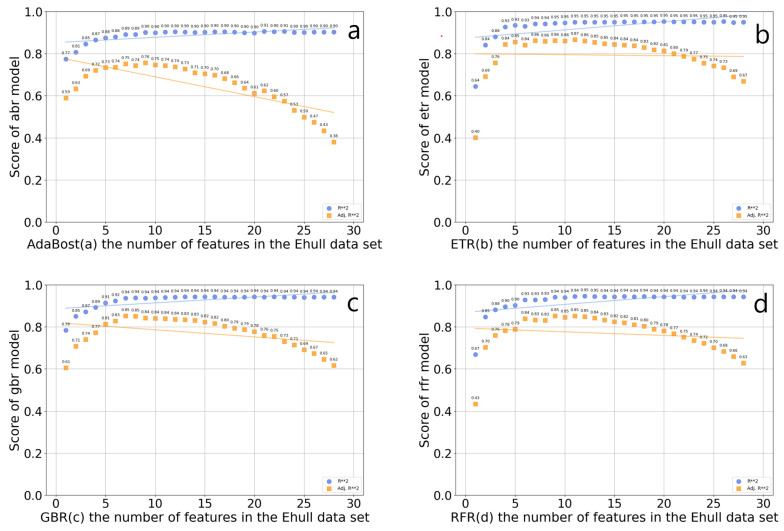
Scores of the four algorithms AdaBoost (**a**), ETR (**b**), GBR (**c**), and RFR (**d**) on the number of features in the E_hull_ data set.

Feature screening is a continuous iterative process, which needs to consider the correlation between data features and target variables, and between features. If multiple features are highly correlated, consider selecting only one of them to reduce redundant information. This can reduce the dimension of the input feature matrix, help screen out important features related to the thermodynamic phase stability of organic–inorganic hybrid perovskites, and shorten the training data time. Four feature selectors were established based on the GBR algorithm, the RFR algorithm, the ETR algorithm, and the AdaBoost algorithm, which were used for feature screening. The worst feature was discarded each time. The score of the four algorithms on the number of features in the data set is shown in Figure 4. In the AdaBoost regression model, the optimal R^2^ value is 0.905936, and the number of features is 17, which includes rx, va, lv, XB_s, XB_pm, X_atoms_any_organic_neighbor_ percent_stdev, X_atoms_any_organic_ neighbor_percent_mean, A_dp, B_atom_X_ neighbor_ percent, C_bp, wc, Ec, A_em, B_atom_surface_area, B_atom_volume, B_atom_globularity, X_atoms_volumes_mean, B_ti, tau, Tf, and Of. In the ETR model, the optimal R^2^ value is 0.951990, and the number of features is 17, which includes rx, lv, XB_s, X_atoms_H_neighbor_percent_stdev, X_atoms_any_organic_neighbor_percent_stdev, X_atoms_any_organic_ neighbor_percent_mean, A_dp, C_bp, wc, Ec, A_em, B_atom_surface_area, B_atom_volume, B_atom_globularity, X_atoms_volumes_mean, B_ti, and Of. In the GBR model, the optimal R^2^ value is 0.943720, and the optimal number of features is 14, which includes rx, va, lv, X_atoms_any_organic_neighbor_percent_stdev, X_atoms_any_organic_neighbor_percent_mean, C_bp, wc, Ec, A_em, and B_atom_volume, B_atom_ globularity, X_atoms_volumes_mean, B_ti, and Of. In the RFR model, the optimal R^2^ value is 0.94585, and the number of features is 12, which includes rx, lv, X_atoms_any_organic_neighbor_percent_stde, X_atoms_any_organic_neighbor_ percent_mean, C_bp, wc, Ec, and B_atom_surface_area, B_atom_globularity, X_atoms_volumes_mean, B_ti, and Of. Among these algorithms, the RFR regression algorithm has the highest optimal R^2^ in the feature screening. Among the above four algorithms, the optimal R^2^ value of ETR is the highest, but the complexity of the ETR model is small, which is prone to underfitting problems. At the same time, the feature subset after ETR screening is input into the prediction model of LightGBM. The MSE and MAE of the prediction model are, respectively, 0.0681 and 0.1886, which are significantly higher than those obtained by using the features selected by random forest as inputs. So, the 12 features of the RFR algorithm are selected as feature subsets, and optimally divide the data set into an 8:2 test set and a training set.

### 2.2. Model Prediction Results

To mitigate dimensional disasters and ensure effective training of the model without over-fitting, the number of descriptors will be constrained to a quantity smaller than the data set’s sample size. Simultaneously, during the hyperparameter tuning process, the model’s performance is evaluated across varying ratios of training and test set, while 10-fold cross-validation is to mitigate issues of under-fitting and over-fitting.

The performance of the prediction model utilizing four machine learning algorithms, RFR, SVR, XGBoost, and LightGBM, is shown in Table 1. The R^2^ of the LightGBM regression model is 0.953914, which measures the proportion of variance between the predicted value and the true value. The R^2^ value ranges from 0 to 1, and the closer it approaches 1, the higher the predictive power of the model. The MAE value is more sensitive to small prediction errors, while the MSE value is more sensitive to large prediction errors. The MAE value of the LightGBM regression is 0.1664, which is the minimum value among the four algorithms. And the MSE value of the LightGBM regression is 0.0531, which is also the smallest among the four models and is closest to 0. The MAE and MSE values indicate that the LightGBM regression model exhibits a smaller prediction error.

**Figure 5:**
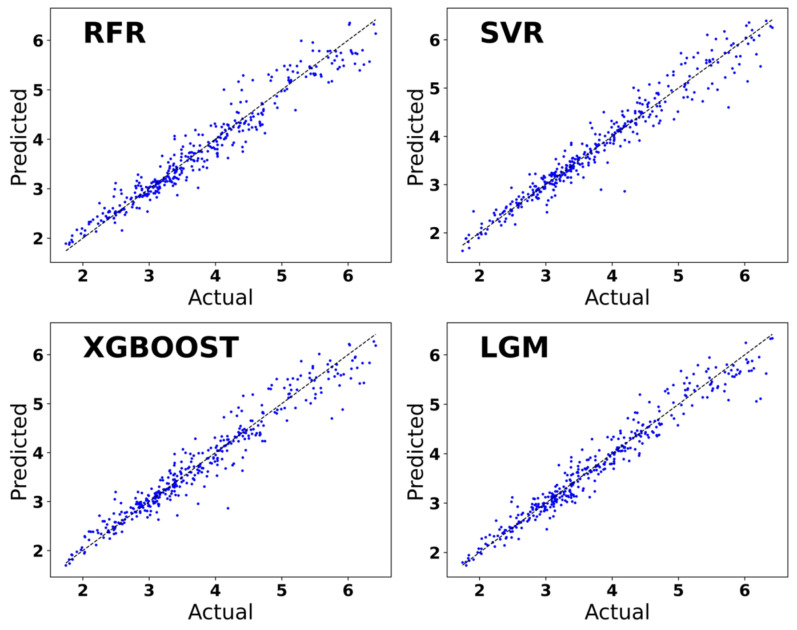
Prediction of E_hull_ used regression models based on the machine learning algorithm.

The training set was trained using four machine learning algorithms, RFR, SVR, XGBoost, and LightGBM. In Figure 5, the horizontal coordinate is the real values of E_hull_, which are obtained through DFT calculations and derived from the data set. The vertical coordinate is the E_hull_ value predicted using machine learning. The prediction of the E_hull_ value of organic–inorganic hybrid perovskite based on the four machine learning algorithms of RFR, SVR, XGBoost, and LightGBM tends to be a straight line with the E_hull_ value of organic–inorganic hybrid perovskite calculated by DFT. It can be concluded that these four algorithms are good at predicting and fitting the E_hull_ value of organic–inorganic hybrid perovskites. The predictive model established by the LightGBM algorithm is better than the SVR algorithm, the RFR algorithm, and the XGBoost algorithm, and the accuracy of the LightGBM model is also greater. The LightGBM regression prediction model emerges as the optimal choice for accurately predicting the E_hull_ values of organic–inorganic hybrid perovskites.

Since the LightGBM prediction model has achieved the best performance in the prediction of E_hull_ for organic–inorganic hybrid perovskites, only the first 20 samples of its test results are recorded in Table 2 for the sake of space.

### 2.3. Interpretation of Prediction Model by SHAP Method

The SHAP method is invaluable for model interpretation and feature selection. It can also serve to validate the accuracy and reliability of the model, identify the weaknesses of the model, and propose improvement schemes. From the interpretation diagram of the SHAP model, the contribution degree of each feature to the prediction results, and the direction and degree of influence of the feature value can be understood. The input feature subsets of the prediction model established by the LightGBM algorithm are rx, lv, X_atoms_any_organic_neighbor_percent_stdev, X_atoms_any_organic_neighbor_percent_mean, C_bp, wc, Ec, B_atom_surface_area, B_atom_globularity, X_atoms_volumes_mean, B_ti, and Of, which are picked out by the RFR algorithm. The SHAP method decomposes the contribution degree of each feature to the prediction result into the sum of the contribution degree of different feature subsets.

**Figure 6:**
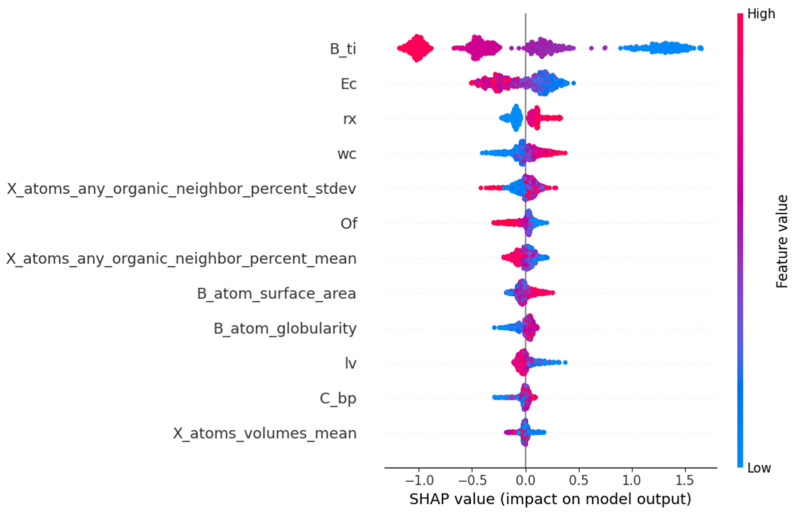
The feature importance ranking of stability for organic–inorganic hybrid perovskites obtained by SHAP value.

In Figure 6, the SHAP values of 12 features are listed in the order of the importance of features from high to low. The horizontal coordinate represents the SHAP value, which is the contribution of each feature to the model’s prediction results. A negative value indicates that the feature is negatively correlated with the prediction results of E_hull_, while a positive value indicates that the feature is positively correlated with the prediction results of E_hull_. The significance of features can be assessed based on the absolute magnitude of the SHAP value. The scatter points in the data set samples represent different observations, with blue dots indicating smaller eigenvalues and red dots representing larger eigenvalues. From Figure 6, the third ionization energy of the B element is the most critical feature in the prediction of E_hull_, and the second key feature is the electron affinity of ions at the X site. They are significantly negatively correlated with the predicted values of E_hull_. The octahedral factors, the average percentage of atoms in the X position adjacent to any atom in the A position, crystal length, and mean volume of the atom at the X position are negatively correlated with predicted E_hull_. The radius of the atom at the X position, atomic weight at the X position, and surface area of the atom at the B position are positively correlated with the predicted E_hull_. In addition, the sphericity of the B atom and boiling point at the X position are also positively correlated with the predicted E_hull_. The magnitude of the SHAP value indicates the relative importance of the corresponding feature, signifying its dominant role in predicting E_hull_. A large value of E_hull_ indicates that the organic–inorganic perovskites are less stable. So, when the values of the third ionization energy of the B element, the electron affinity of ions at X position, the average percentage of atoms in X position adjacent to any atom in A position, crystal length, and mean volume of the atom at X position are larger, the thermodynamic phase stability of perovskites is higher. When the values of the radius of the atom at the X position, atomic weight at the X position, the surface area of the atom at the B position, the sphericity of the B atom, and boiling point at the X position are smaller, the stability of organic–inorganic perovskites is higher. In physics, the third ionization energy is the energy at which an atom’s electrons are separated from its nucleus between two ionization levels. It significantly indicates chemical properties, including the tendency of high-energy ion reactions, molecular stability, and conformational transformations. The higher the ionization energy of an atom, the greater its structural stability. Among the results of prediction, the third ionization energy of the B element and electron affinity of ions at the X site are the two most critical characteristics. It is indicated that LightGBM regression is feasible to predict the thermodynamic stability of perovskites, and the prediction of E_hull_ can assist in verifying the structural stability obtained by tolerance factor analysis.

## 3. Data and Methods

### 3.1. Data Sources

**Figure 7:**
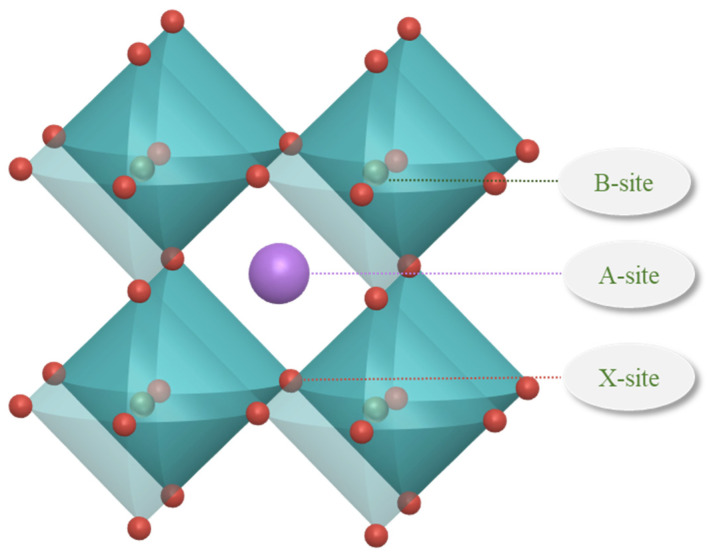
Structure diagram of perovskites.

Raw data sets include 1254 raw data, which come from the Materials Project, an open access materials database (established by Lawrence Berkeley National Laboratory and Carnegie Mellon University), and some of the literature [30,37]. The structure of perovskites is typically characterized by octahedral arrangements, as illustrated in Figure 7. The ions at the A site, B site, and X site contained in the data set are detailed below. The ions at the A site are organic amine cations; the ions at the B site are metal cations of the elements Ge, Pb, and Sn; and the ions at the X site are anions of the elements C, Br, I, and Cl. The feature descriptors of the thermodynamic phase stability prediction for organic–inorganic hybrid perovskites are seen in Table 3. The data set was randomly divided into an 80% training set and a 20% validation set to train and validate the machine learning model.

### 3.2. Machine Learning Algorithms and Model Evaluation

#### 3.2.1. Gradient-Boosting Regression

Gradient-boosting regression (GBR) is an ensemble learning algorithm. It combines multiple weaker learning algorithms, typically decision trees, to create a more powerful model for improving prediction accuracy [38]. The algorithm flow of GBR is as follows: through continuous iteration, multiple weak regression models are combined to form a strong model.

Let the training data set be $\left\{\left(x_{1},y_{1}\right),\left(x_{2},y_{2}\right),\cdots ,(x_{n},y_{n})\right\}$, where $x_{i}\in Rm,$ yi∈R,m is the characteristic number. First, initialize the model according to the following function:

(3) $$f_{0}(x)=argmin_{\gamma }\sum_{i=1}^{n}L(y_{i},\gamma )$$

For each iteration step *t* = 1, 2, *...*, *T*, calculate the negative gradient by Formula (4); by fitting the residual, the leaf node region of the *t*-th tree is obtained, and the output value of each leaf node is calculated by Formula (5). The model is then updated, as shown in Formula (6), to return the final model $f_{T}(x)$.

(4) $$r_{it}=-[\frac{\partial L(y_{i},f(x_{i}))}{\partial f(y_{i})}{]}_{f(x)=f_{t-1}(x)}$$

(5) $$\gamma_{jt}=argmin_{\gamma }\sum_{x_{i}\in R_{jt}}L\left(y_{i},f_{t-1}\left(x_{i}\right)+\gamma \right)$$

(6) $$f_{t}(x)=f_{t-1}(x)+\sum_{j=1}^{J}\gamma_{jt}I(x\in R_{jt})$$

where L(y,f(x)) is the loss function, I(x∈Rj) is the indication function. In the GBR algorithm, the square error loss function is usually used.

#### 3.2.2. Random Forest Regression

Random forest regression (RFR) is also an ensemble learning algorithm. It constructs multiple decision trees to generate predictions and subsequently combines the predictions from each tree to form the final outcome [39]. The random forest regression algorithm can effectively deal with the over-fitting problem and improve the prediction ability of the model. The proposed algorithm exhibits superior performance in handling high-dimensional and nonlinear data compared to conventional regression methods. The proposed method simultaneously achieves high accuracy and robustness, thereby effectively shortening the development cycle and reducing costs in the material development process.

The main steps of the random forest regression algorithm are as follows. (1) Select *p* samples from the training set by a “self-help method” to form a training set subset as a new training set. (2) When constructing the decision tree, if each sample has *K* attributes, then *K* (*k* less than *K*) attributes are randomly selected from these *K* attributes during the splitting process of each node. Then, according to the variance of these k attributes, one is selected as the best split attribute of the node. (3) During the generation of the decision tree, the splitting of each node is performed iteratively according to step 2 until further splitting becomes infeasible. The decision tree is trained by using this subset, and no pruning is performed on this decision tree. (4) Build a large number of decision trees according to steps 1–3 until *m* decision trees are trained. (5) For each test sample, regression prediction is performed individually by every decision tree, followed by statistical analysis of the prediction results from all decision trees for the same sample. The final prediction value is obtained by calculating the average of these results.

#### 3.2.3. Extra Trees Regression

The extra trees regression algorithm (ETR) is mainly used to solve regression problems [40]. The core idea of the algorithm is to obtain the final predicted value by constructing multiple decision trees and a weighted average of the results of each decision tree. In the process of constructing each decision tree, the ETR regression algorithm randomly selects a part of the samples and a feature subset of the training set for training to minimize the error. It can process the missing data, and optimize the model parameters by using the self-help method and cross-validation, so as to improve the accuracy and generalization ability of the model.

#### 3.2.4. Support Vector Regression

Support vector regression (SVR) [41] is a regression algorithm based on support vector machines. In contrast to conventional regression algorithms, SVR does not directly predict the value of the target variable; instead, it optimizes the margin between training data points to identify a function that can yield superior predictions for unknown data. According to the actual problem, the algorithm can improve the generalization ability of the model by adjusting the parameters of the kernel function and penalty function. Support vector regression algorithm performs better than traditional machine learning algorithms in handling experimental or observational data with low noise, especially in machine learning with small sample data.

#### 3.2.5. Adaptive Boosting 

The AdaBoost algorithm (AdaBoost) is an ensemble learning method based on the boosting strategy, which trains weak learners sequentially in a highly adaptive way [42]. Its core idea is to adjust the weight of error samples, and then upgrade iteratively.

AdaBoost’s regression algorithm process is as follows.

(1) Initialize the weights of each sample (all weights are equal) by the Formula (7).

(2) Calculate the error rate. According to the calculation formula of the error rate (see Formula (8)), construct the weak learner with the smallest error rate.

(3) Adjust the weight of the weak learner to obtain the strong learner of the m iteration.

(4) Increase the weight of incorrectly classified samples and reduce the weight of correctly classified samples, so that more accurate classification can be achieved later.

(5) Iterate over the above process until the error score reaches a threshold or the number of iterations reaches a set maximum. The expression of the final strong learner obtained after several iterations is shown in Formula (9).

(7) $$w_{mi}=\frac{1}{N}\left(i=1,2,\ldots ,N\right)$$

(8) $$e_{m}=\sum_{i=1}^{N}\;w_{mi}I\left(F_{m}\left(x_{i}\right)\neq y_{i}\right)$$

(9) $$sign[f_{M}\left(x\right)]=sign[\sum_{i=l}^{M}\;a_{i}F_{i}\left(x\right)]$$

where the error rate $e_{m}$ is the sum of the weights of the misclassified samples; the predicted value $F_{m}\left(x_{i}\right)$ refers to the classification of the sample *i* predicted by the weak learner $F_{m}\left(x\right)$; $w_{mi}$ is the weight of the sample; $y_{i}$ is the actual value; $I\left(F_{m}\left(x_{i}\right)\neq y_{i}\right)$ is the indicating function, whose value is 1 if the prediction fails and the condition in parentheses holds, and, otherwise, whose value is 0. And where $sign\left(x\right)$ is the symbolic function, as shown in the following formula.

(10) $$sign\left(x\right)=\left\{\begin{array}{r} 1,\;x>0\\ 0,\;x=0\\ -1,\;x<0 \end{array}\right.$$

#### 3.2.6. Extreme Gradient Boosting (XGBoost)

The extreme gradient-boosting (XGBoost) algorithm [43] is an algorithm implementation based on the boosting framework. It conforms to the form of a model function, and the output of the model can be expressed as the sum of the output of *K* weak learners. The basic idea of the algorithm is to reduce the bias of the model by constantly generating new trees, each tree learning based on the difference between the previous tree and the target value to reduce the bias of the model. The output of the final model result is shown in the Formula (11). That is, the results of all trees add up to what the model predicts for a sample.

(11) $${\hat{y}}_{i}^{\left(t\right)}=\sum_{k=1}^{i}f_{k}\left(x_{i}\right)={\hat{y}}_{i}^{\left(t-1\right)}+f_{t}\left(x_{i}\right)$$

where ${\hat{y}}_{i}^{\left(t\right)}$ is the prediction result of the sample *i* after the *t*-th iteration, ${\hat{y}}_{i}^{\left(t-1\right)}$ is the prediction result of the front *t* − 1 tree, and $f_{t}\left(x_{i}\right)$ is the model of the *t*-th tree.

The objective function consists of the loss function of the model and the regular term that inhibits the complexity of the model, and the formula is as follows.

(12) $$Obj=\sum_{i=1}^{n}l(y_{i},{\hat{y}}_{i})+\sum_{i=1}^{t}\Omega (f_{i})$$

where $\sum_{i=1}^{n}l(y_{i},{\hat{y}}_{i})$ is to sum the complexity of all trees and it is added to the objective function as a regularization term to prevent the model from over-fitting.

#### 3.2.7. Lightweight Gradient Lifting Algorithm (LightGBM)

The lightweight gradient lifting algorithm (LightGBM) is a machine learning algorithm based on a gradient lifting decision tree (GBDT) developed by the Microsoft team [44]. The LightGBM uses a histogram-based decision tree algorithm and mutually exclusive feature bunding to process a large number of data instances and a large number of features, which improves the training efficiency and accuracy of the model. Compared with the traditional GBDT algorithm, the LightGBM has higher accuracy and faster training speed.

Suppose the data set is $H=\{(x_{i},y_{i}){\}}_{i=1}^{n}$, use the LightGBM algorithm to find the proximate value f(x) of a function $\hat{f}\left(x\right)$, and minimize the loss function L(y,f(x)) through the function. To judge the quality of the model fitting data, observe the size of the loss function, whose optimization function can be expressed as follows:

(13) $$\begin{array}{c} \hat{f}=\arg\;\underset{f}{min}E_{y,X}L\left(y,f(x)\right) \end{array}$$

Meanwhile, the LightGBM model integrates *k* regression trees to fit the final model, which can be expressed as follows:

(14) $$f_{k}\left(x\right)=\sum_{i=1}^{k}\;f_{t}\left(H\right)$$

### 3.3. Evaluation Indicators of Model

The performance of regression models is evaluated using the following metrics: mean squared error (MSE), mean absolute error (MAE), and R^2^. The MSE measures the sum of squares of the difference between the predicted value and the true value, and the MAE is the average of the difference between the predicted value and the true value, which are indicators used to measure forecasting error [45,46]. But, the difference is that MSE is more sensitive to larger prediction errors, while MAE is more sensitive to smaller prediction errors [47]. The R^2^ value is another commonly used metric that measures how well the model explains changes in the data, representing the degree of linear correlation between the regression value and the true value. The value of R^2^ ranges from 0 to 1, and a higher R^2^ indicates a better model explaining changes in the data.

Their formulas are as follows:

(15) $$MSE=\frac{1}{m}\sum_{i=1}^{m}(f_{i}-y_{i}{)}^{2}$$

(16) $$MAE=\frac{1}{m}\sum_{i=1}^{n}\left|y_{i}-{\hat{y}}_{i}\right|$$

(17) $$R^{2}=1-\frac{\sum_{i=0}^{n-1}(y_{i}-f_{i}{)}^{2}}{\sum_{i=0}^{n-1}(y_{i}-{\bar{y}}_{i}{)}^{2}}$$

where *m* is the number of samples; $f_{i}$ is the true value; $y_{i}$ is the predicted value.

## 4. Conclusions

In this work, the original data set of E_hull_ for organic–inorganic hybrid perovskites were constructed using crystal structure data and material composition, and the data were preprocessed by exploratory data analysis. And the Pearson correlation coefficient analysis was employed to compare the original features, select effective features as feature subsets, and optimally divide the data set into an 8:2 ratio for testing and training purposes. Various machine learning prediction models were established using the four kinds of machine learning algorithms: RFR, SVR, XGBoost, and LightGBM, to predict the E_hull_ of organic–inorganic hybrid perovskites. The results show that the prediction model of the LightGBM algorithm has the best prediction effect with a MAE value of 0.1664, a MSE value of 0.0531, and an R^2^ value of 0.953914. The model exhibits a low prediction error, which indicates that the input features fit the prediction model well. Compared with the original input features and default parameters, the performance of the model is greatly improved. The prediction results of the LightGBM model were analyzed by the SHAP value, and it was found that the third ionization energy of B element and electron affinity of ions at X site contributed more to the stability prediction and they are significantly negatively correlated with the prediction results of E_hull_ value. A large value of E_hull_ indicates that the organic–inorganic perovskites are less stable. The stability of perovskites is closely related to its crystal structure. The higher values for the third ionization energy of the B element and the electron affinity of ions at the X site may help maintain the stability of the crystal structure. In the screening of organic–inorganic perovskites with high stability, priority can be given to the third ionization energy of B element and electron affinity of ions at the X site. The results are expected to provide guidance for the design and synthesis of novel organic–inorganic hybrid perovskites and provide references for machine learning prediction research of other materials.

In the future, various methods should be tested to reduce multicollinearity, such as principal component analysis (PCA), and data enhancement techniques for limited data sets will be explored, in order to improve the accuracy of the machine learning model. Moreover, machine learning and DFT methods will be used to select candidate element combinations with high thermodynamic phase stability.

## Figures and Tables

**Table 1:** Comparison of performance outcomes from regression models.

Algorithm	Evaluating Indicator		
	MAE	MSE	R^2^
RFR	0.1884	0.0662	0.942552
SVR	0.1713	0.0614	0.946759
XGBoost	0.1839	0.0689	0.940207
LightGBM	0.1664	0.0531	0.953914

**Table 2:** Prediction results of the LightGBM model for E_hull_ on the test set.

HOIP Order Number	E_hull_ (eV/Oatom)	ML_E_hull_ (eV/Oatom)	Prediction Error
1	3.087	3.070	0.016
2	5.6831	5.309	0.373
3	4.3624	4.366	−0.003
4	4.6596	5.407	−0.747
5	3.6561	3.704	−0.048
6	5.039	5.743	−0.704
7	3.5853	3.764	−0.179
8	5.8538	5.801	0.051
9	3.196	3.727	−0.531
10	4.0485	3.933	0.115
11	2.9874	3.007	−0.020
12	5.8294	5.823	0.006
13	4.622	4.419	0.202
14	3.5053	3.530	−0.025
15	4.9335	4.754	0.179
16	2.6572	2.531	0.125
17	5.1533	4.809	0.344
18	2.7138	2.542	0.171
19	2.9218	3.237	−0.315
20	1.9719	2.0300	−0.058

**Table 3:** Feature descriptors of E_hull_ for organic–inorganic hybrid perovskites.

Feature	Unit	Description
A site	None	Chemical formula for the ion at A site
B site	None	Chemical formula for ions at B site
X site	None	Chemical formula for ions at X site
rb	Å	Radius of the ion at B site
ra	Å	Radius of the ion at A site
tf	None	Tolerance factor
lv	Å	Crystal length
XB_s	Å	Proportion of B and X atoms standard deviation
XB_pm	None	Average percentage of adjacent X and B atoms
X_atoms_H_neighbor_percent_stdev	None	Percentage standard deviation of adjacent atoms in X position
X_atoms_volumes_stdev	None	Standard deviation of volume for X atom
X_atoms_H_neighbor_percent_mean	Å	Average percentage of adjacent X atom
X_atoms_any_organic_neighbor_percent_stdev	None	Percentage standard deviation of X and A positions
X_atoms_any_organic_neighbor_percent_mean	Å	Average percentage of atoms in X position adjacent to any atom in A position
A_dp	C/m^2^	Dipolarizability
B_atom_X_neighbor_percent	Å	Percentage of atoms at B position to X position
C_bp	°C	Boiling point at X position
wc	Å	Atomic weight at X position
Ec	eV	Electron affinity of ions at X position
A_em	Å	Lattice constant of ions at A position
B_atom_surface_area	Å^3^	Surface area of atom at B position
B_atom_volume	Å^3^	Volume of atom at B position
B_atom_globularity	°	Sphericity of the B atom
X_atoms_volumes_mean	Å	Mean volume of the atom at X position
X_atoms_volumes_stdev	Å^3^	Standard deviation of the volume for atom at X position
of	None	Octahedral factors
rx	Å	Radius of atom at X position
tau	None	New tolerance factor
B_ti	eV	Third ionization energy of B element
B_lc	nm	Lattice constant of B element
B_ep	None	Crystal structure at B element

## Data Availability

Data are contained within the article.
